# Address

**Published:** 1866-04

**Authors:** J. Chesebrough


### AN ADDRESS.

*Opening Address before the Mississippi Valley Dental Association, by the President,* Dr. J. Chesebrough, *Feb.* 21, 1866.

*Gentlemen:*

Another year has passed away, and the events which have transpired within it have been forgotten or become matters of history.

Since our last meeting the world has seen pass before it such a panorama as will never be seen again. The curse of this land has been at one stroke removed, and the rebellion smitten, both never to rise again.

We are now in peace; and in that peace abounding in prosperity. Have we not much to give thanks for at this satisfactory state of affairs at this present day.

While feeling that our national affairs assume such a pleasing shape, we have great cause for thankfulness that our professional ones are none the less so. At no time since the foundation of our specialty have we felt the hope and the satisfaction that we do at this day. Our army of laborers have fought their way over many a rough road, bridged many a chasm, fought manfully for the right, and now knowing what is right, dare maintain it.

Look backward, if you will, for a little, and see from what we sprang, and, coming forward through the intervening time witness where we now stand. One hundred years ago where were all the aspirations, or even the nucleus of the science of to-day. Let us quietly drop the curtain over these ancient years and ways, they brighten not our hopes for the future in the recollection of the past. In regard to them let the motto ever be "*requiescat in pace.*" We may drop out the intervening half century ere we arrive at the progressive age, or the beginning of it. Within this period down to within twenty-five years, comparatively little was done. We must not forget the researches of the indefatigable Lewenhoek nearly two centuries ago, or later of John Hunter and Thomas Bell, those beacon lights that now shine out boldly amid the darkness.

But beyond these and other few, we have nothing upon which to boast at the beginning of a half century ago. The labors of our profession at that day, comprised little more than the extraction of teeth; in fact the Dentist, as such, was an unheard of being. The Barber then was the Dentist of the place, and frequently its Surgeon. Those indeed were dark days, to consign the sufferings of humanity into such murderous hands.

How each one of us would shrink from such hands were we the victims, in those days, of one of the thousand ills arising from the disorganization of the teeth.

It is said that our specialty is but little more than thirty years old. We can safely assume that age for it, for its growth can be put almost within those bounds. And what a growth!

From the puny offspring, willing to be fathered by none, looked upon by its brothers, Medicine and Surgery, as an illegitimate, it has grown to manhood, received the acknowledgement of equality from all its relations. Acknowledged by *itself* and accepted as a tie binding Medicine and Surgery, making a triplet with the unit and doublet.

To whom shall we accord the measure of praise due for all this; not only to Lewenhoek, and Hunter, Bell, Tomes, Nasmyth, Muller, Goodsir, Owen, Harris, Kolliker, Bond, but to those of our age and generation who have staked their all, and an indefatigable will, that success should be the crown.

We owe it to enterprise and ambition, and a determination to suceed at all hazards. Look if you will at the operations of thirty years ago and of to-day, and who among us would now countenance those first efforts? Who would for any remuneration go back to those days, and carve the denture from the ivory when, through the perseverance and will of Allen, we have at our hands, almost, if not an equal of nature?

Who would take the first effort of Stockton, when by the indefatigability of White, we are the possessors of imitation, that to all intents and purposes is a reality? And who among us would transfer the point and mallet of to-day for the old rope? Who would go backward and accept the doctrine that an aching tooth is a lost one, and therefore must be extirpated, instead of that most excellent practice whereby it is our duty to save all and extirpate none?

If as we look around us, we see such great changes as these; if we behold the light of knowledge shining on and before us; if that radiance which goes before to lighten our pathway, shines still brighter and brighter, day by day illuminating some intricate recess, where we have been stumbling over obstacles, and which are now brought to our sight and comprehension as clear as the light of day, and if we are led to believe that the future is as pregnant as the past, have we *no* reason to feel our hearts expand to comprehend the full measure of all this?

Each day witnesses some change for the better, some improvement which is a step in advance. And what is the cause and where lies the secret of all this progress? I can attribute it to associate effort more than to anything else; to just such meetings as this, where we come up to interchange our ideas, plans and perfections.

And the reason why such meetings tend to advance is that our natural contraction has to suffer an expansion. We hear the views and experience of others, and wherein we can put them into practice.

We find out thereby *that something* which we have long been seeking for, and it at once enters that storehouse of our minds, to come out again at a future day improved. We find that knowledge can only be gained by study, and our determination not to be in the rear rank urges us to renewed action as we pursue our vocation.

We come here and find out the immensity of the field of our specialty, and if we intend to be true and faithful to our patients and ourselves, we must study well all those intricacies of disease, which have their basis in the disorganization of the teeth, or are in sympathy with them.

We dive into the unfathomable depths of this sea; at whose bottom we are sure to find those pearls of great value to reward us for our labors.

The higher tone of the discussions that take place at these meetings, fit us better for our labors, and the more exalted nature of the each succeeding year, is another proof of our advancement.

Look if you will, at the order of exercises, and the lay out of subjects for the discussion at this meeting, and compare it with ten years ago. Then the half of the time of a meeting, was devoted to the pros and cons of amalgam, or similar nonality, now we strike infinitely higher and strive to see what burning flames we can bring out of Materia Medica, Physiology and Pathology.

We are but just upon the confines of the "promised land," and our own endeavors will carry us further into it. My heart beats with pleasure, as I dare give the brain freedom to run over a space of years, and behold this profession at the end of ten or twenty years from now.

The vividness of imagination is inadequate to foretell what may be in those days.

Had any one dared to foretell, fifty years ago, the advance of to-day, he would have been pronounced a maniac. I will not weary you with any picture I might paint; I could not give it too much color, however vividly I might portray the advance that will surely be made in that space of time. When we have so many true, earnest workers in the field, we may almost count upon anything as the result of those days.

Gentlemen, for you and me it is a pleasure to live in these days, more so if we are able by our research to advance our profession and receive the merit thereof.

This association having now passed its maturity, should be second to none. Born twenty-two years ago it is now the oldest in existence. It has outlived all others of its day. It has seen in some a death from inactivity, a rusting out; in others it has seen the bone of contention gradually swallow them up. It has seen the resurrection of others to life again after years of deathlike sleep. It has been at the birth of many, and extended always a cordial greeting to all its associates. Its latch-string has never been drawn within the door, and can be always found hanging on the outside.

It has been noted for its enterprise, its liberality and its advancement. It has never been in the rear rank. It always intends to be in the advance. Its actions are quoted over the East, West, North and South, where there is suffering humanity to relieve.

Your energies must be taxed to keep it in the advance guard. Your record must be placed among its archives, in order to hold up its arms in the battle for the right. I can do but little cf myself; with your assisstance I may do much. Mine I freely give you, that we may be as a bundle of rods, giving strength to each other and not to be easily burst asunder. Let us, therefore, take fresh courage, and strive to the utmost to advance not only ourselves, but our neighbors and friends, that we may be proud of our parentage, and make the name of the Old Mississippi Valley ring cheerfully in every ear.

